# Supplementary material for: Are Methylation Patterns in the KALRN Gene Associated with Cognitive and Depressive Symptoms? Findings from the Moli-sani Cohort
Source: Int J Mol Sci. 2024 Sep 25;25(19):10317. doi: 10.3390/ijms251910317 (PMC11476580; doi:10.3390/ijms251910317)
Supplement: Supplementary file 1 [file ijms-25-10317-s001.zip › ijms-3163296-supplementary.pdf]

Supplementary Methods

Targeted DNA pyrosequencing and quality control (QC)

1,474 samples underwent targeted DNA pyrosequencing of the region chr3:124584826-124584886. This region was selected since it was in the promotor of the (brain-specific) isoform 2 of the *KALRN* gene (*Gencode Transcript: ENST00000682363.1*), hence methylation patterns in this region may have a potential regulatory role on the expression of the gene.

DNA extraction was performed through a Salting Out method following these steps: 10 mL of Red Cell Lysis (RCL) buffer were added to blood sample and were incubated on ice for 15’, followed by a centrifuge at 4°C, 2000 rpm, for 15’. Then, supernatant was discarded, and this step was repeated adding 7 mL of RCL buffer to wash the pellet. 500 µL of Salt-EDTA (SE) buffer, 10 µL of Proteinase K 10 mg/ml and 50 µL of SDS 10% were added to pellet. After an over-night incubation at 37°, 200 µL of NaCl 5M were added to each sample and shaken for 15’ at room temperature followed by a centrifuge at 4°C, 4000 rpm for 15’. Supernatant was transferred to a new 2 mL Eppendorf tube. After repeating this step, 800µL of Isopropanol were added to each sample and gently mixed until DNA strand appeared. The DNA strand was taken out with a closed glass Pasteur pipet and transferred in 70% ice cold ethanol to wash it. DNA was transferred to Eppendorf tube, let dry for about 10’ and finally eluted in 150 µL of Tris-EDTA (TE) buffer 1X.

Then we checked the amount of DNA obtained and its quality using MaestroNano Micro-Volume Spectrophotometer (Maestrogen). 95% of DNA samples had a good-quality with $A_{260/A280}$ ratio of 1.7–2.0 and a $A_{260/A230}$ ratio of 2.0-2.2. Moreover, we performed a further QC of DNA through agarose gel electrophoresis and all samples were found to be of good quality.

To randomize the samples to 96-wells plates, we used an in-house built R script [1] based on the sample function [1], without replacement, controlling for sex and age ratios across plates to avoid excessive departures from the total sample (1.36 and 63.46, respectively).

Then we performed a bisulfite treatment of DNA using the EZ-96 DNA Methylation Kit (ZYMO RESEARCH®), according to the manufacturer's instructions.

Our assay consists of 3 primers:

- Forward: 5’-GGT AGT GGT TTT TAG TAA GTT AGA GTT-3’
- Reverse: Biotin 5’-ACA ACC ACT CAC CTC TAC AC-3’
- Sequencing: 5'-GGG GAG GAG GGG TAA TAT GA-3'

These were designed using PyroMark Assay Design Software 2.0 and one of the two PCR amplification primers (the reverse primer) was biotinylated. PCR was performed using the PyroMark PCR Kit following the manufacturer's instructions and the following conditions: 15’ at 95°C (denaturation), 15 sec at 95°C, 30 sec at 62°C and 30 sec at 72°C for 45 cycles (DNA amplification) and final extension 10’ at 72°C. Pyrosequencing reaction was conducted on the PyroMark Q48 instrument according to the manufacturer’s instructions. Through the sequencing primer we analyzed the region chr3:124584826-124584886 (hg38), identifying three different CpGs, located in chr3:124584830, chr3:124584833 and chr3:124584849-C-G, also known as rs56407180. Samples were randomized across 16 plates, each including 3 positive controls: 1 completely methylated bisulfite converted DNAs, 1 completely unmethylated bisulfite converted DNAs, 1 untreated unmethylated genomic DNA (EpiTect PCR Control DNA Set, Qiagen©), and 1 negative control.

DNA methylation analysis through Illumina EPIC array

1,099 samples were extracted as above and analyzed through the Illumina EPIC array (v1, 865,918 CpGs), in the context of a project investigating epigenetic changes associated with polypharmacy in elderly.

97% of DNA samples had a good-quality with A_{260}/A_{280} ratio of 1.7–2.0 and a A_{260}/A_{230} ratio of 2.0-2.2, assessed through MaestroNano Micro-Volume Spectrophotometer (Maestrogen). Again, we performed a further QC of DNA through agarose gel electrophoresis of a random subset (10%) of samples. All samples tested showed good quality.

To randomize the samples to 96-wells plates, we used an in-house built R script based on the sample() function in R [1] , without replacement, controlling for sex and case-control ratios across plates to avoid excessive departures from the total sample (1.05 and 2.64, respectively). Then we performed a bisulfite treatment of DNA using the EZ-96 DNA Methylation Kit, Cat #D5003 (ZYMO RESEARCH®) to convert unmethylated cytosines to uracils, according to the manufacturer's instructions. Bisulfited DNA was

used for DNA methylation evaluation through Human EPIC Bead Chip (Illumina®) according to manufacturer's instructions. The methylation experiment was performed by the Genomic Core Facility at ARGO Open Lab Platform for Genome sequencing, AREA Science Park, (TS, Italy). To estimate the methylation status, the Illumina Infinium assay utilizes a pair of probes (methylated and unmethylated probe) to measure the intensities of the methylation at the interrogated CpG site. The methylation level is then estimated based on the measured intensities of this pair of probes. This value ranges from 0 (unmethylated) to 1 (fully methylated site).

QC of methylation signals was carried out in ChAMP v2.20.1 [2], an integrated analysis pipeline for epigenome-wide association scans. Probes were filtered out if they showed a detection P > 0.01, fewer than 3 beads in more than 5% of all samples; if they were cross-reactive probes; if they were located on sex chromosomes or if they showed biased DNA methylation signal due to Single Nucleotide Polymorphisms detected at CpG sites. Overall, the totality of samples (1,162) and 668,413 probes passed epigenome-wide QC and computed beta values underwent i) a beta-mixture quantile (BMIQ) normalization, ii) identification of batch effects through singular value decomposition (svd) to test associations between principal components biological and technical factors, and iii) combat analysis to remove such effects (technical variables adjusted included: plate, well, slide and array). Finally, these were converted to M-values. From the resulting probes passing QC, we rescued 137 candidate CpGs out of 165 total probes annotated to the *KALRN* gene, based on the EPIC BeadChip manifest (reported in Table S1), to analyze the association with methylation patterns across the whole gene and not only in a targeted region.

Covariates

Sociodemographic data were obtained by interviewer-administered questionnaires. Education was based on the highest qualification attained and was categorized as up to primary (≤ 5 years of study), lower secondary (≤ 8 years), upper secondary school (>8≤13 years) and postsecondary level (>13 years). Data on pre-existing chronic diseases and health conditions were also collected. Personal history of cardiovascular disease (angina, myocardial infarction, revascularization procedures, peripheral artery diseases and cerebrovascular events) was self-reported and confirmed by medical records – as was

history of cancer - and therapy in use. Participants were considered to have diabetes or hyperlipidemia if they were taking disease-specific drugs, as assessed by the interviewer.

Subjects were also classified as never, current, or former smokers (reported not having smoked at all over the previous 12 months or more).

Body mass index (BMI) was calculated as weight divided by squared height (kg/m^2).

Habitual food intake was assessed through the semi-quantitative EPIC food frequency questionnaire (FFQ), validated and adapted to the Italian population [3] to evaluate the participants' diet in the previous 12 months. The FFQ includes a total of 188 foods that were classified into 45 predefined food groups based on similar nutritional characteristics or culinary use. Using specially designed software [4] the frequencies and quantities of each food were linked to the Italian Food Tables [5] to obtain estimates of the daily intake of macro and micronutrients and energy. We asked participants to indicate the number of times they consumed a particular food (per day, week, month, or year), from which we calculated the absolute frequency of consumption of each food. We assessed the amount of food consumed by asking the participant to select an image of a portion of food or a predefined standard portion when no image was available [6]. For ethanol, men consuming 10-50 g/day and women consuming 5-25 g/day received 1 point; otherwise, 0 points were assigned.

Adherence to the traditional MD was determined through the MD score developed by Trichopoulou et al. [7] and was obtained by assigning 1 point to healthy food groups - including fruits and nuts, vegetables, legumes, fish, cereals, monounsaturated (MUFA) to saturated fatty acids (SFA) ratio - whose consumption was above the sex-specific medians of intake of the Moli-sani study population. Conversely, foods presumed to be detrimental like meat and dairy products were scored positively if their consumption was below the median. The MD score ranged from 0 to 9 with increasing values reflecting maximal adherence.

DIS (dietary inflammation score) and LIS (lifestyle inflammatory score) were calculated using the method described by Byrd et al. [8]. For DIS, 19 food groups (18 whole foods and beverages and 1 composite micronutrient supplement group) were selected *a priori* based on biological plausibility and previous literature (Table S2). The DIS components were acquired from FFQ used in our cohort [3] and weights were developed assessing the strengths of the multivariable-adjusted associations of each component with a panel of circulating inflammatory biomarkers – including high-sensitivity C-reactive

protein, interleukins 6, 8 and 10 – as computed in [8]. An individual's DIS or LIS was then calculated as the sum of their weighted components.

LIS included four components: smoking status, physical activity, alcohol intake, and BMI, with weights determined as above. Because the weights were developed based on cross-sectional exposure–biomarker associations, smoking was categorized as “current” or “former/never.” BMI (kg/m^2) was categorized as underweight/normal (<25), overweight (25–29.99), or obese (≥30). Heavy alcohol consumption was defined as > 1 or > 2 drinks (>14 or >28 g of ethanol, respectively)/day for women and men, respectively; moderate consumption was defined as consumed alcohol but in less than these amounts. Physical activity was categorized as the frequency of being physically active enough to work up a sweat (0, 1–3, or ≥ 4 times/week), a previously well-validated measure [9] [10].

Further analysis of depressive symptoms

To explore the significant associations detected between depressive symptoms and KALRN epigenetic variants (see main text), we carried out further association analyses testing factors underlying common variance shared among depressive symptoms and single PHQ9 items as outcomes.

First, we performed a polychoric factor analysis of all the symtpoms assessed through PHQ9, namely i) Little interest or pleasure in doing things (anhedonia); ii) Feeling down, depressed, or hopeless (low mood); iii) Trouble falling or staying asleep, or sleeping too much (altered sleeping); iv) Feeling tired or having little energy (tiredness, low energy); v) Poor appetite or overeating (altered appetite/eating); vi) Feeling bad about yourself – or that you are a failure or have let yourself or your family down (feeling of failure); vii) Trouble concentrating on things, such as reading the newspaper or watching television (impaired concentration); viii) Moving or speaking slowly, or alternatively being fidgety and restless (altered movements/speaking); ix) Thoughts that you would be better off dead or of hurting yourself in some way (suicidal ideation). To this end, we used the psych package (https://cran.r-project.org/web/packages/psych/index.html), first computing a polychoric correlation matrix among all symptoms (Figure S2a), then computing the resulting factors through oblimin rotation and analyzing the loadings of each depressive factor extracted (Figure S2b). These revelead the existence of two main factors, one with higher loadings of cognitive symptoms (MR1, explaining 27% of total shared variance) and one clearly tagging somatic symptoms (MR2, explaining 30% of total variance). These factors showed a high correlation (Pearson’s r = 0.81). Each of the two factors resulting from the analysis was

then analyzed as outcome, in multivariable regression models incrementally adjusted for all the covariates used in the main analyses, as well as for the other factor not used as an outcome, so to detect associations which were not driven by the variance shared among the depressive domains they represented.

Similarly, we dichotomized each single PHQ9 item analyzed into two different categories: participants reporting that a specific symptom occurred at least "more than half of the days" were classified as "affected" for the specific item (1), otherwise they were classified as "not affected" (0). Each (dichotomous) symptom was then tested in multivariable logistic regressions vs the candidate CpG tested, adjusted for age, sex, education level, prevalent health conditions (CVD, diabetes, dyslipidemia, cancer, BMI) and lifestyles like LIS, DIS and MD scores, smoking, alcohol (g/day) and energy intake (Kcal/day). Moreover, in each model all the other items excluding the one used as the outcome were used as covariates to residualize the associations which were shared with other symptoms, as well as cognitive performance (MoCA score).

Figure S1. Histograms of methylation levels of the three CpGs analyzed through pyrosequencing.

a)

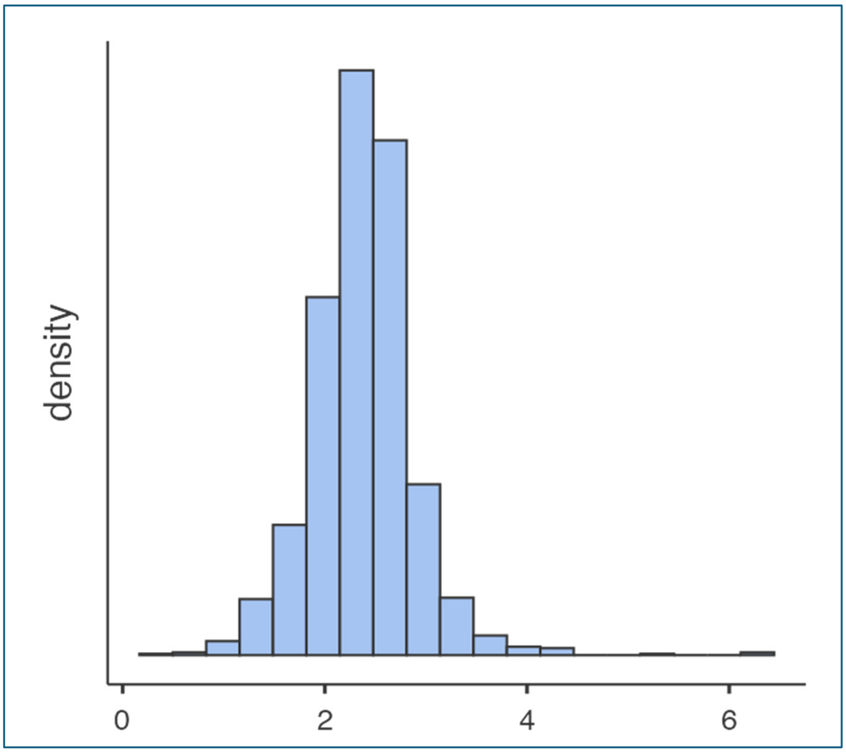

b)

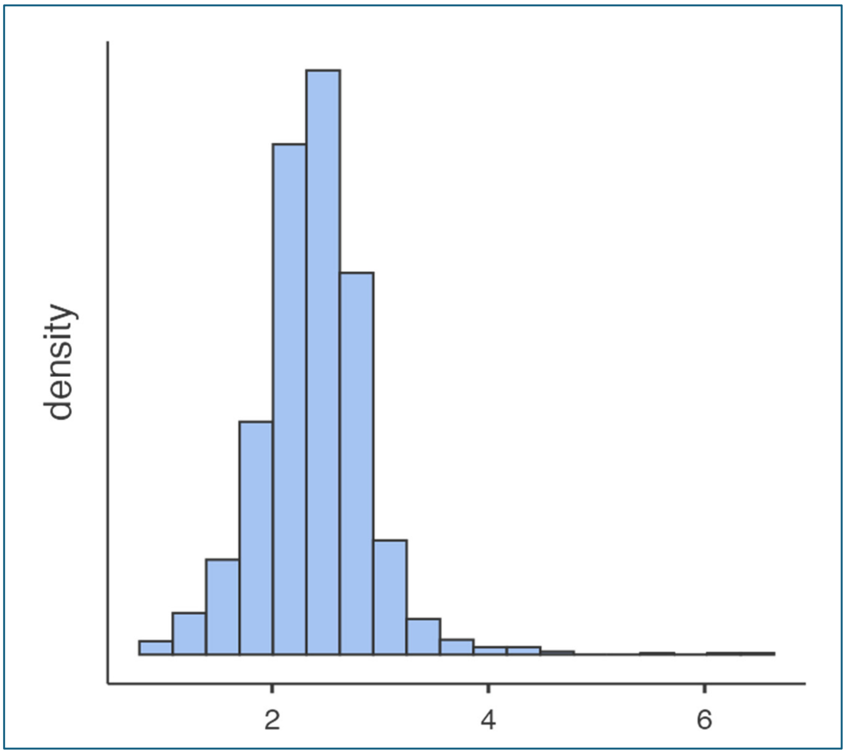

c)

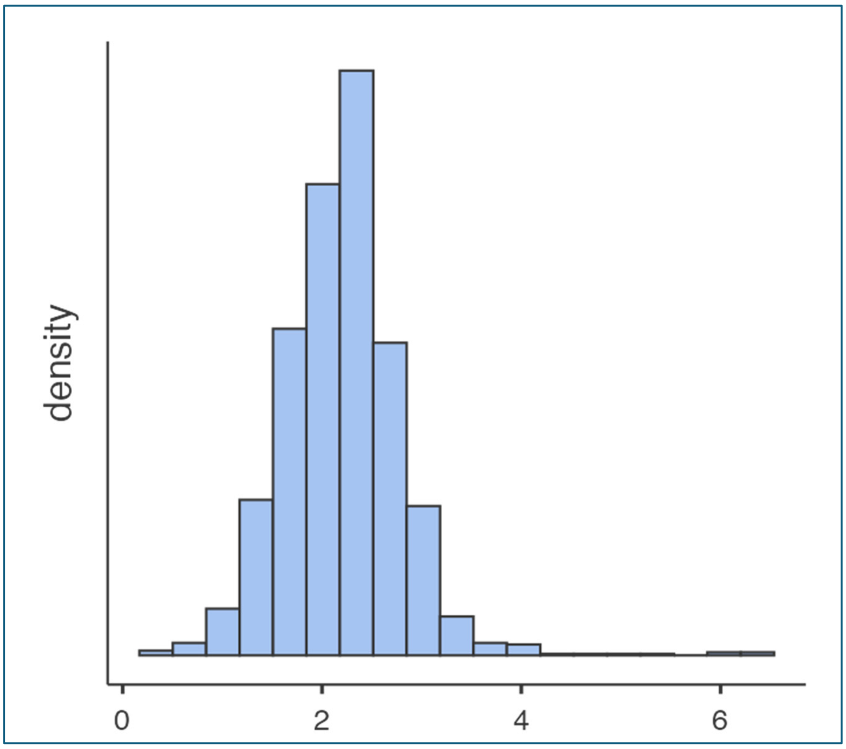

Log2 values of the methylation levels are reported for CpG1 (chr3:124584830), CpG2 (chr3:124584833) and CpG3 (chr3:124584849-C-G, hg38).

Table S1. KALRN probes available in Illumina® EPIC Bead chip.

CpG	Position(GRCh37)	Position(GRCh38)	Analyzed
cg13036944	123797420	124078573	no
cg22305574	123797483	124078636	no
cg26905399	123798063	124079216	no
cg05720721	123803128	124084281	yes
cg05121182	123812381	124093534	yes
cg08577025	123812517	124093670	yes
cg27617244	123813090	124094243	yes
cg19928195	123813191	124094344	yes
cg13151664	123813232	124094385	yes
cg12144803	123813383	124094536	yes
cg25184248	123813393	124094546	yes
cg13308143	123813419	124094572	yes
cg11939346	123813454	124094607	yes
cg24829557	123813465	124094618	yes
cg24323597	123813504	124094657	yes
cg16278716	123813587	124094740	yes
cg10430690	123813596	124094749	yes
cg13549966	123825585	124106738	yes
cg07736222	123830489	124111642	yes
cg15956635	123831798	124112951	yes
cg08011218	123834913	124116066	yes
cg07586984	123838429	124119582	no
cg00198586	123861151	124142304	yes
cg05584242	123866984	124148137	yes
cg11148677	123876715	124157868	yes
cg26510634	123877804	124158957	yes
cg03660538	123894269	124175422	no
cg05633656	123916684	124197837	yes
cg10908929	123927041	124208194	yes
cg09109865	123946703	124227856	yes
cg21618455	123950018	124231171	yes
cg13560249	123962200	124243353	yes
cg26655180	123965948	124247101	yes
cg21425296	123967656	124248809	yes
cg21726846	123968973	124250126	yes
cg11336987	123974913	124256066	yes
cg00924106	123976875	124258028	yes
cg12024811	123979446	124260599	yes
cg20130098	123984695	124265848	no
cg01718380	123985521	124266674	yes
cg08383262	123987194	124268347	yes

cg04816013	123987591	124268744	yes
cg19918027	123987645	124268798	yes
cg20714128	123987727	124268880	no
cg07011093	123987823	124268976	yes
cg22376833	123987916	124269069	yes
cg05766129	123988013	124269166	no
cg02593416	123988666	124269819	yes
cg10464130	123991925	124273078	yes
cg10165543	123996394	124277547	yes
cg20097985	124003076	124284229	yes
cg00259083	124017729	124298882	yes
cg00086941	124034100	124315253	yes
cg05056583	124036925	124318078	yes
cg13002109	124041146	124322299	no
cg24257704	124053151	124334304	yes
cg09211564	124053570	124334723	yes
cg16100687	124066633	124347786	yes
cg20570458	124069933	124351086	yes
cg24252694	124070437	124351590	yes
cg02118562	124082907	124364060	yes
cg24954696	124087201	124368354	no
cg16992277	124092133	124373286	yes
cg23949492	124101967	124383120	yes
cg21640650	124102766	124383919	no
cg10178498	124103021	124384174	yes
cg04807106	124103560	124384713	yes
cg07225857	124103650	124384803	yes
cg05469885	124103652	124384805	yes
cg23440058	124103696	124384849	yes
cg27647255	124115408	124396561	yes
cg03508390	124120796	124401949	yes
cg14958919	124125583	124406736	yes
cg21083798	124125950	124407103	yes
cg13779109	124126494	124407647	yes
cg07745037	124126573	124407726	no
cg00984696	124128381	124409534	no
cg11229104	124132963	124414116	yes
cg07414162	124146219	124427372	yes
cg17016275	124148564	124429717	yes
cg26162691	124170647	124451800	yes
cg24118591	124173868	124455021	yes
cg08003225	124186888	124468041	yes
cg07483657	124187430	124468583	yes

cg14521373	124195489	124476642	yes
cg10103145	124206959	124488112	yes
cg26931898	124221765	124502918	yes
cg07618561	124222817	124503970	yes
cg07600936	124222879	124504032	yes
cg01474669	124222910	124504063	yes
cg16229398	124223488	124504641	yes
cg17479671	124226331	124507484	no
cg13098874	124227120	124508273	no
cg06690085	124232190	124513343	no
cg01092804	124232294	124513447	yes
cg16919675	124237310	124518463	yes
cg13689591	124239629	124520782	no
cg09751451	124240030	124521183	yes
cg17045232	124245663	124526816	yes
cg12210841	124269432	124550585	yes
cg04833271	124271103	124552256	yes
cg06153410	124276792	124557945	yes
cg23475018	124277638	124558791	yes
cg21167201	124277912	124559065	yes
cg11463851	124278885	124560038	yes
cg16598299	124281086	124562239	yes
cg09990141	124281984	124563137	yes
cg08789152	124282341	124563494	yes
cg02764852	124282766	124563919	yes
cg06218523	124284219	124565372	yes
cg24421473	124284458	124565611	no
cg02456998	124293098	124574251	yes
cg24253342	124298415	124579568	yes
cg20237595	124301252	124582405	yes
cg23981549	124302724	124583877	yes
cg14688451	124303035	124584188	yes
cg06205331	124303268	124584421	yes
cg13648874	124303351	124584504	yes
cg22422051	124303360	124584513	yes
cg20925263	124303389	124584542	no
cg04516112	124303404	124584557	yes
cg23837547	124303408	124584561	yes
cg18981420	124303541	124584694	yes
cg10338082	124303555	124584708	yes
cg02754399	124303564	124584717	yes
cg19522900	124303745	124584898	yes
cg14874541	124304033	124585186	yes

cg24175649	124304161	124585314	yes
cg04915618	124304960	124586113	no
cg15001930	124306820	124587973	no
cg22139363	124312243	124593396	no
cg09757846	124312317	124593470	yes
cg07965493	124320000	124601153	yes
cg15272979	124326148	124607301	yes
cg14559160	124327006	124608159	no
cg06143364	124330501	124611654	yes
cg01734829	124340057	124621210	yes
cg09229974	124342971	124624124	no
cg13261226	124343102	124624255	no
cg12777296	124343115	124624268	yes
cg09899430	124354118	124635271	yes
cg04468444	124354566	124635719	no
cg00593835	124355674	124636827	yes
cg27633580	124355701	124636854	yes
cg26052377	124356493	124637646	yes
cg18807940	124358702	124639855	yes
cg15438184	124358997	124640150	yes
cg03883133	124369830	124650983	yes
cg07966155	124373219	124654372	yes
cg11688731	124376592	124657745	yes
cg21274175	124378446	124659599	yes
cg02290008	124382944	124664097	yes
cg24354101	124383535	124664688	yes
cg16395286	124393169	124674322	yes
cg18042859	124393413	124674566	yes
cg05980111	124395277	124676430	yes
cg03572074	124395533	124676686	yes
cg22389313	124396052	124677205	yes
cg11815961	124398309	124679462	yes
cg15285869	124408043	124689196	yes
cg02293874	124408740	124689893	yes
cg01738411	124417054	124698207	no
cg02423740	124418957	124700110	yes
cg20807374	124420060	124701213	yes
cg26849111	124438600	124719753	no

Table S2. Components, descriptions, and weights of the components of the dietary (DIS) and lifestyle (LIS) inflammation scores in the Moli-sani study.

Components	Descriptions	Weights
DIS components[1]		
Leafy greens and cruciferous vegetables	Lettuce, broccoli, Brussels sprouts, cauliflower, turnip green, cabbage, kale, and spinach	-0.14
Tomatoes	Tomatoes, and tomato sauce	-0.78
Other vegetables	Peppers, onions, artichokes, celery, mushrooms, eggplant, and beets	-0.16
Apples and berries	Apple, pear, strawberries, and cherry	-0.65
Deep yellow or orange vegetables and fruit	Peach, cooked and row carrots, squash, and figs	-0.57
Other fruits and real fruit juices	Bananas, kiwi, oranges, grapefruits, mandarins, grapefruit, plums, melon, kaki, fruit juices, and orange juices	-0.16
Legumes	Beans, lentils, and chickpeas	-0.04
Fish	Shellfish, shrimps and crustaceous, dried fish (i.e. salted codfish), canned fish and other fish not included in the previous categories (i.e. salmon, swordfish, anchovies, etc.)	-0.08
Poultry	Chicken or turkey with and without skin	-0.45
Red and organ meats	Hamburger, beef, pork, mutton, lamb, and offal	0.02
Processed meats	Canned meat, and processed meat	0.68
High-fat dairy	Whole and semi-skimmed milk, whole milk, fruit yogurt, ice cream and hard cheese.	-0.14
Low-fat dairy	Low-fat cheese, ricotta cheese, and low-fat yogurt.	-0.12
Added sugars	Chocolate, candies, dried fruits, jams, canned fruits, dry cakes, sweetened carbonated beverages, and non-carbonated fruit drinks	0.56
Coffee and tea	Coffee (decaffeinated and caffeinated), and tea	-0.25

Refined grains and starchy vegetables	Breakfast cereals, bread, rice, pasta, potatoes, and home-baked or ready-made cake	0.72
Nuts	Walnuts, hazelnuts, almonds, and peanuts	-0.44
Fats	Butter, gravy, margarine, and mayonnaise or other creamy dressing	0.31
Supplement score[2]	Ranked score of supplements, including: vitamins A, B, C, D, multivitamins, B-complex vitamins and iron	-0.80
LIS components[3]		
Current smoker	Currently smoked tobacco at baseline vs. does not currently smoke tobacco	0.50
Heavy drinker	Heavy [> 1 drink (> 14 g ethanol)/d for women, > 2 drinks (28 g ethanol)/d for men] vs. nondrinker.	0.30
Moderate drinker	Moderate [1 drink (14 g ethanol)/d for women, 1–2 drinks or (14–28 g ethanol)/d for men] vs. nondrinker.	-0.66
Heavily physically active	Exercises ≥ 4 times/week vs. does not exercise	-0.41
Moderately physically active	Exercises 1–3 times/week vs. does not exercise	-0.18
Obese BMI	Obese BMI (≥ 30 kg/m^2) vs. normal/underweight BMI (< 25 kg/m^2)	1.57
Overweight BMI	Overweight BMI (]25 – 30[kg/m^2) vs. normal/underweight BMI (< 25 kg/m^2)	0.89

[1]Dietary components were standardized by sex, to a mean of zero and SD of 1.
[2]All individual supplements were dummy variables, coded as “1” for consumption category and “0” for no-consumption for anti-inflammatory supplements (vitamins A, B, C, D, multivitamins, B-complex vitamins) and “0” or “-1” for pro-inflammatory supplements (iron).
[3]All lifestyle components were dummy variables, coded as 1 for the non-referent category and 0 for the referent category.

Table S3. Baseline characteristics of the population under study.

Variable	Sub-cohort undergoing pyrosequencing (N = 1,385)	Sub-cohort analyzed through EPIC array (N = 1,024)	P for difference
Sex (women, %)	58.0	50.7	<0.001
Age (y)	63.1 (9.07)	71.3 (5.37)	<0.001
Education (%)			
Primary or less	8.3	16.3	<0.001
Lower secondary	24.0	20.6	
Upper secondary	46.4	42.7	
Post-secondary	21.2	20.4	
Health conditions (%)			
CVD	8.6	8.6	1.00
Diabetes	11.0	8.5	0.04
Dyslipidemia	24.5	20.7	0.03
Cancer	9.2	7.9	0.28
BMI	28.2 (4.94)	28.1 (4.46)	0.71
Lifestyles and other factors			
LIS_SCORE	0.49 (0.75)	0.47 (0.73)	0.54
DIS_SCORE	-63.0 (111)	-73.2 (107)	0.02
Smokers (current/previous, %)	17.3/31.6	13.3/40.8	<0.001
MEDscore	4.35 (1.59)	4.50 (1.60)	0.02
Alcohol intake (g/day)	9.52 (14.5)	13.7 (18.5)	<0.001
PHQ9	4.13 (4.09)	3.83 (3.79)	0.07
MoCA	24.8 (3.32)	23.8 (3.49)	<0.001
Energy intake (Kcal/day)	1835 (534)	1809 (490)	0.22

We report frequency (%) for categorical variables, or, alternatively, mean values and standard deviations (SD) for continuous variables. The population undergoing pyrosequencing and the one involved in the EPIC array analysis are independent. P-values resulting from statistical comparisons of these sub-cohorts– carried out through Mann-Whitney U test for continuous variables and Chi-Squared test for categorical variables - are rounded to the second decimal place, unless they were significant.

Abbreviations: CVD, cardiovascular disease; BMI, body mass index; LIS_score (lifestyle inflammatory score); DIS_score (dietary inflammation score); MEDscore, adherence score to Mediterranean Diet; (PHQ9), Patient Health Questionnaire 9; (MoCA), The Montreal Cognitive Assessment.

Supplementary Results

Figure S2. Polychoric factor analysis of the nine depressive symptoms assessed through PHQ9.

a)

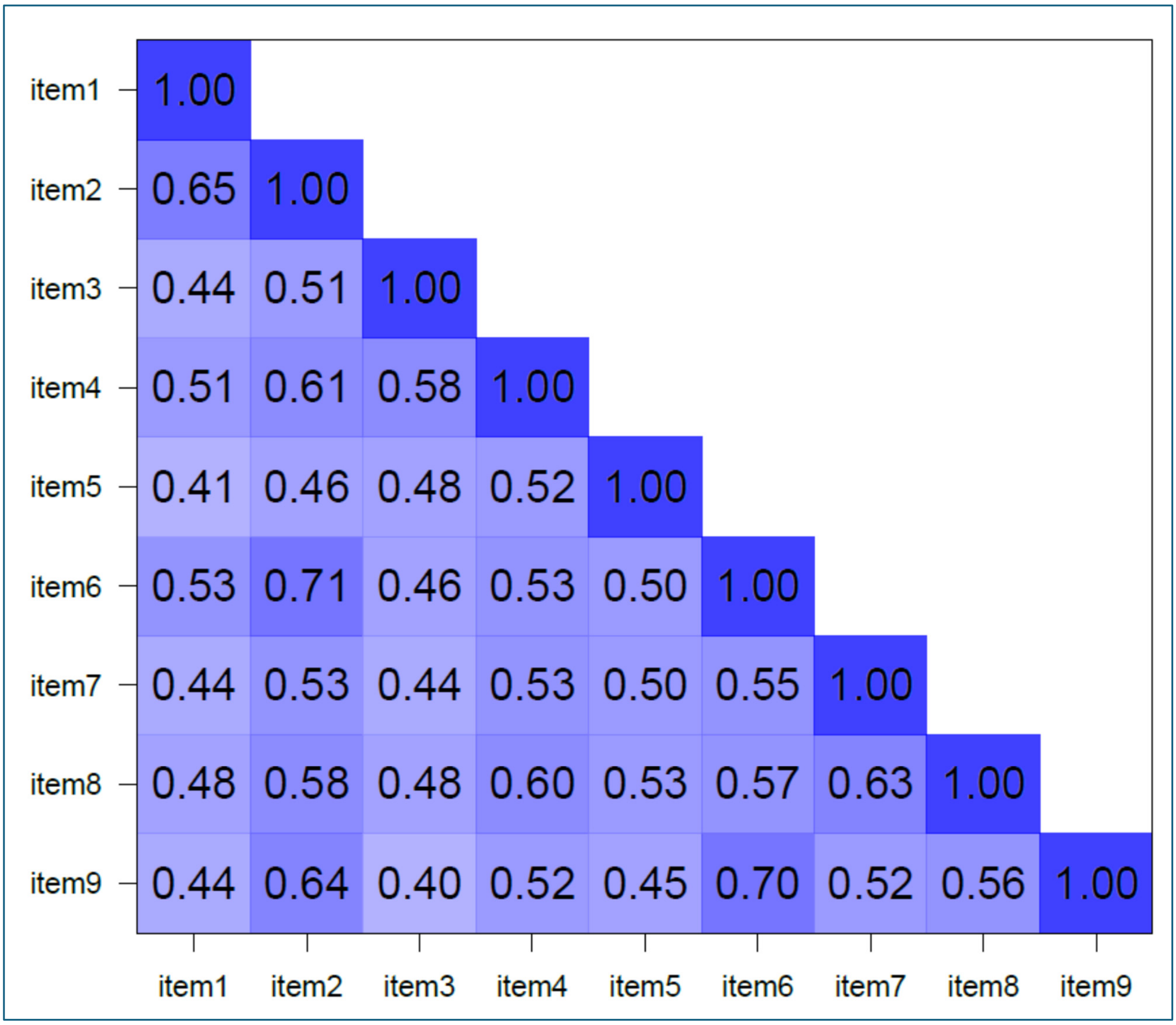

b)

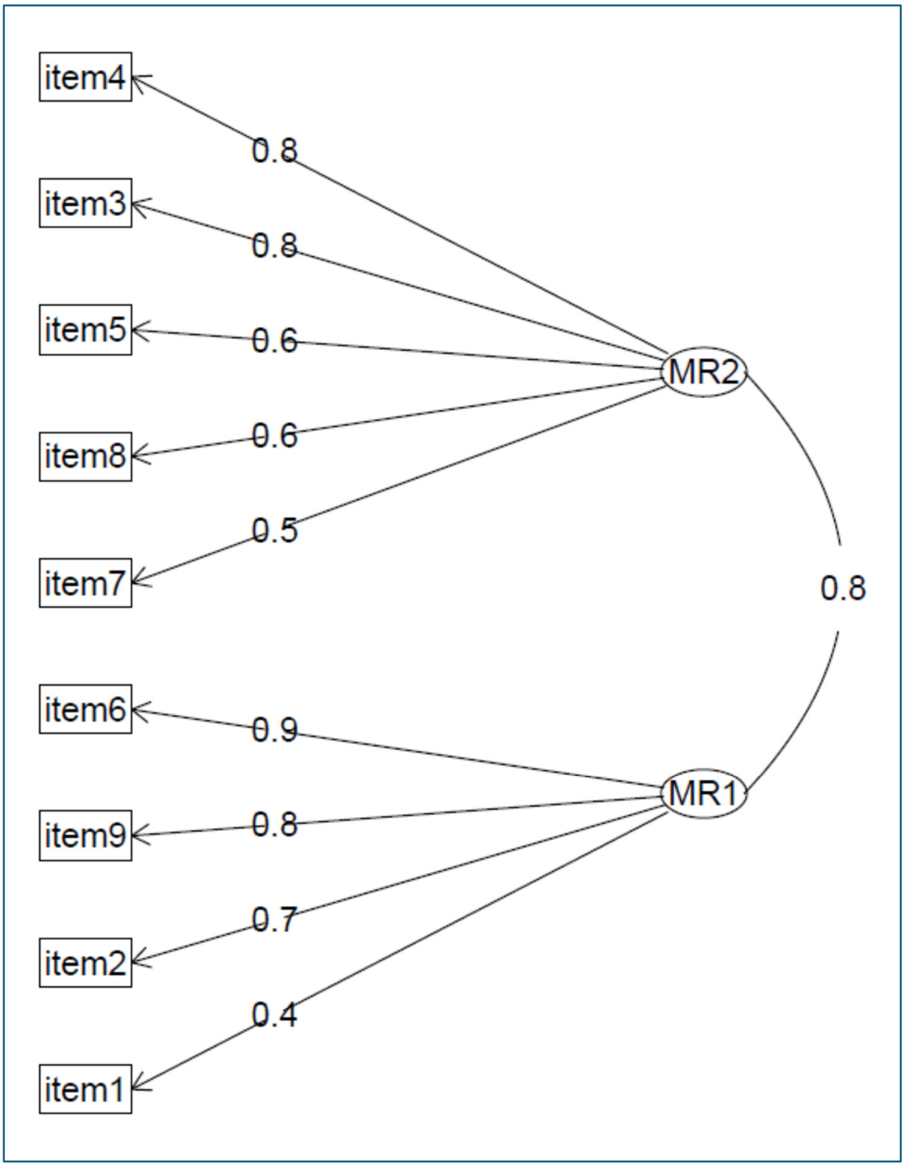

Polychoric **a)** correlation matrix and and **b)** factors extracted from the nine items of the PHQ9 scale are reported, along with loadings on each factor (with absolute values greater than 0.3). Factors showed a Pearson correlation of 0.8 (since they were extracted through oblique rotation).

Legend: MR1 = "somatic" factor; MR2 = "cognitive" factor;. item 1 = anhedonia; item 2 = low mood; item 3 = altered sleeping; item 4 = tiredness, low energy; item 5 = altered appetite/eating; item 6 = feeling of failure; item 7 = impaired concentration; item 8 = altered movements/speaking; item 9 = suicidal ideation.

Table S4. Results of the association analysis of cognitive performance (MoCA) and depressive symptoms (PHQ9) vs methylation levels of the three CpGs tested.

Regression Model	CpG1<br>β (SE), p	CpG2<br>β (SE), p	CpG3<br>β (SE), p
**MoCA**	0.071 (0.285), 0.80	-0.175 (0.283), 0.54	0.138 (0.203), 0.50
**PHQ-9**	-0.272 (0.407), 0.51	0.477 (0.404), 0.24	0.057(0.290), 0.85

Association Beta and relevant Standard Errors (β(SE)) of the fully adjusted regression model including all the CpGs together are reported, along with (raw) p-values. Adjustment: age + sex + lymphocytes (%) + monocytes (%) + granulocytes (%) + education level + prevalent health conditions (cvd, diabetes, dyslipidemia, cancer, BMI) + lifestyles (LIS, DIS and MD scores, smoking, alcohol (g/day) and energy intake (Kcal/day)) + MoCA or PHQ9 (depending on the outcome). Abbreviations: PHQ9 = Patient Health Questionnaire 9; MoCA), = The Montreal Cognitive Assessment.

Table S5. Results of the analysis of the polychoric factors extracted from depressive symptoms vs methylation levels of cg13549966.

Regression Model	MR1<br>β (SE), p	MR2<br>β (SE), p
**Model 1**	-0.068 (0.064), 0.288	0.123 (0.064), 0.055
**Model 2**	-0.075 (0.064), 0.247	0.129 (0.064), 0.044
**Model 3**	-0.069 (0.064), 0.286	0.123 (0.064), 0.053
**Model 4**	-0.073 (0.064), 0.253	0.123 (0.064), 0.053
**Model 5**	-0.072 (0.064), 0.262	0.127 (0.064), 0.048

Association Beta and relevant Standard Errors (β(SE)) of the incrementally adjusted regression models are reported, along with p-values, for the polychoric factors tagging cognitive (MR1) and somatic (MR2) depressive symptoms.
Legend: Model 1: age + sex + lymphocytes (%) + monocytes (%) + granulocytes (%); Model 2: Model 1 + education level; Model 3: Model 2 + prevalent health conditions (CVD, diabetes, dyslipidemia, cancer, BMI); Model 4: Model 3 + lifestyles (LIS, DIS and MD scores, alcohol (g/day) and energy intake (Kcal/day); Model 5: Model 4 + MoCA score. Note: all the models were adjusted for the polychoric factor other than the one used as outcome.

Table S6. Associations of the different depressive symptoms vs methylation levels of cg13549966.

PHQ9 item	Depressive symptom	cg13549966 β (SE), p
1	Little interest or pleasure in doing things (anhedonia)	0.047 (0.027), 0.08
2	Feeling down, depressed, or hopeless (low mood)	-0.012 (0.022), 0.60
3	Trouble falling or staying asleep, or sleeping too much (altered sleeping)	0.062 (0.038), 0.10
4	Feeling tired or having little energy (tiredness, low energy)	0.018 (0.031), 0.56
5	Poor appetite or overeating (altered appetite/eating)	0.005 (0.027), 0.85
6	Feeling bad about yourself – or that you are a failure or have let yourself or your family down (feeling of failure)	0.027 (0.020), 0.18
7	Trouble concentrating on things, such as reading the newspaper or watching television (impaired concentration)	-0.010 (0.025), 0.68
8	Moving or speaking so slowly that other people could have noticed? Or the opposite – being so fidgety or restless that you have been moving around a lot more than usual (altered movements/speaking)	-0.022 (0.024), 0.35
9	Thoughts that you would be better off dead or of hurting yourself in some way (suicidal ideation)	-0.009 (0.013), 0.48

Association Beta and relevant Standard Errors (β(SE)) of the fully adjusted regression model are reported, along with p-values. Adjustment: age + sex + lymphocytes (%) + monocytes (%) + granulocytes (%) + education level + prevalent health conditions (CVD, diabetes, dyslipidemia, cancer, BMI) + lifestyles (LIS, DIS and MD scores, smoking, alcohol (g/day) and energy intake (Kcal/day)) + MoCA score + all the other items excluding the one used as the outcome.

Moli-sani Study Investigators

The enrolment phase of the Moli-sani Study was conducted at the Research Laboratories of the Catholic University in Campobasso (Italy), the follow up of the Moli-sani cohort is being conducted at the Department of Epidemiology and Prevention of the IRCCS Neuromed, Pozzilli, Italy.

Steering Committee: Licia Iacoviello*#(Chairperson), Giovanni de Gaetano*, Maria Benedetta Donati*.

Scientific Secretariat: Chiara Cerletti* (Coordinator), Marialaura Bonaccio*, Americo Bonanni*, Simona Costanzo*, Amalia De Curtis*, Augusto Di Castelnuovo§, Alessandro Gialluisi*#, Francesco Gianfagna§°, Mariarosaria Persichillo*, Teresa Di Prospero* (Secretary).

Safety and Ethical Committee: Jos Vermylen (Catholic University, Leuven, Belgio) (Chairperson), Renzo Pegoraro (Pontificia Accademia per la Vita, Roma, Italy), Antonio Spagnolo (Catholic University, Roma, Italy).

External Event Adjudicating Committee: Deodato Assanelli (Brescia, Italy), Livia Rago (Campobasso, Italy).

Baseline and Follow-up Data Management: Simona Costanzo* (Coordinator), Marco Olivieri (Campobasso, Italy), Sabatino Orlandi*, Teresa Panzera*.

Data Analysis: Augusto Di Castelnuovo§ (Coordinator), Marialaura Bonaccio*, Simona Costanzo*, Simona Esposito*, Alessandro Gialluisi*#, Anwal Ghulam*, Francesco Gianfagna§°, Roberta Parisi, Antonietta Pepe*, Emilia Ruggiero*, Sukshma Sharma*.

Biobank, Molecular and Genetic Laboratory: Amalia De Curtis* (Coordinator), Concetta Civitillo*, Alisia Cretella*, Sara Magnacca§, Fabrizia Noro*.

Recruitment Staff: Mariarosaria Persichillo* (Coordinator), Francesca Bracone*, Giuseppe Di Costanzo*, Sabrina Franciosa*, Martina Morelli*, Teresa Panzera*.

Communication and Press Office: Americo Bonanni*.

Regional Institutions: Direzione Generale per la Salute - Regione Molise; Azienda Sanitaria Regionale del Molise (ASReM, Italy); Agenzia Regionale per la Protezione Ambientale del Molise (ARPA Molise, Italy); Molise Dati Spa (Campobasso, Italy); Offices of vital statistics of the Molise region.

Hospitals: Presidi Ospedalieri ASReM: Ospedale A. Cardarelli – Campobasso, Ospedale F. Veneziale – Isernia, Ospedale San Timoteo - Termoli (CB), Ospedale Ss. Rosario - Venafro (IS), Ospedale Vietri – Larino (CB), Ospedale San Francesco Caracciolo - Agnone (IS); Casa di Cura Villa Maria - Campobasso; Responsible Research Hospital - Campobasso; IRCCS Neuromed - Pozzilli (IS).

*Department of Epidemiology and Prevention, IRCCS Neuromed, Pozzilli, Italy

#Department of Medicine and Surgery, LUM University "Giuseppe Degennaro", Casamassima, Italy

§Mediterranea Cardiocentro, Napoli, Italy

°Department of Medicine and Surgery, University of Insubria, Varese, Italy

Moli-sani Study Past Investigators are available at https://www.moli-sani.org/?page_id=173

References

1. R Core Team, R., *R: A language and environment for statistical computing.* 2013.
2. Tian, Y., et al., *ChAMP: updated methylation analysis pipeline for Illumina BeadChips.* Bioinformatics, 2017. **33**(24): p. 3982-3984.
3. Pisani, P., et al., *Relative validity and reproducibility of a food frequency dietary questionnaire for use in the Italian EPIC centres.* International Journal of Epidemiology, 1997. **26**(suppl_1): p. S152-S152.
4. Pala, V., et al., *Diet in the Italian EPIC cohorts: presentation of data and methodological issues.* Tumori, 2003. **89**(6): p. 594-607.
5. Palli, D., et al., *A Molecular Epidemiology Project on Diet and Cancer: The Epic-Italy Prospective Study. Design and Baseline Characteristics of Participants.* 2003. **89**(6): p. 586-593.
6. Bonaccio, M., et al., *Joint association of food nutritional profile by Nutri-Score front-of-pack label and ultra-processed food intake with mortality: Moli-sani prospective cohort study.* Bmj, 2022. **378**: p. e070688.
7. Trichopoulou, A., et al., *Adherence to a Mediterranean diet and survival in a Greek population.* N Engl J Med, 2003. **348**(26): p. 2599-608.
8. Byrd, D.A., et al., *Development and Validation of Novel Dietary and Lifestyle Inflammation Scores.* J Nutr, 2019. **149**(12): p. 2206-2218.
9. WASHBURN, R.A., et al., *THE VALIDITY OF SELF-REPORTED EXERCISE-INDUCED SWEATING AS A MEASURE OF PHYSICAL ACTIVITY.* American Journal of Epidemiology, 1990. **132**(1): p. 107-113.
10. Washburn, R.A., L.L. Adams, and G.T. Haile, *Physical activity assessment for epidemiologic research: the utility of two simplified approaches.* Prev Med, 1987. **16**(5): p. 636-46.
